# Development of a low back pain care pathway in an academic hospital system: results of a consensus process

**DOI:** 10.1186/s13018-023-04492-z

**Published:** 2024-01-03

**Authors:** Geronimo Bejarano, Robert Vining, Devan P. Desai, Joe Minchew, H. Michael Guo, Christine Goertz

**Affiliations:** 1 Brown University, Providence, RI USA; 2 Palmer College of Chiropractic, Davenport, IA USA; 3 Duke University, 300 W. Morgan Street, Office 441, Durham, NC 27701 USA

**Keywords:** Low back pain, Clinical practice guideline, Care pathway, Delphi, Chiropractic, Physical therapy, Spinal pain

## Abstract

**Background:**

Low back pain (LBP) is the leading cause of disability worldwide and a significant component of healthcare expenditures. Clinical practice guidelines (CPGs) have been highlighted as a key resource to improve the quality of care. This study aimed to develop a clinical pathway for LBP based on CPGs in an academic health system.

**Methods:**

We conducted a modified Delphi study of clinicians caring for patients with LBP who were asked to rate 21 CPG-informed seed statements through an online survey. The goal was to identify statements that achieved a minimum of 80% consensus among panelists.

**Results:**

Thirty-five healthcare providers participated as panelists. The majority of participants were male (68.6%), had MD or DO (62.9%) degrees, and were clinicians (73.8%) working in neurosurgery (36.1%), orthopedics (25.7%), emergency medicine (14.3%), or physical therapy (11.4%). Initially, consensus was reached on 20 of 21 seed statements. One statement did not reach consensus in the initial round and was revised into two separate statements based on feedback from panelists. One of these statements achieved consensus in the second review round. All statements reaching consensus were incorporated into a care pathway consisting of diagnosis, evaluation, and treatment for LBP.

**Conclusion:**

Healthcare providers across various disciplines supported statements interpreting current CPGs related to care for LBP. This study represents a step toward supporting guideline-concordant care for LBP. Additional research is needed to assess how such pathways impact actual clinical care.

## Background

Low back pain (LBP) is the leading cause of disability worldwide and a major component of healthcare expenditures [1, 2]. In the USA, more is spent on low back and neck pain ($134.6 billion in 2016) than any other condition [3]. Clinical practice guidelines (CPGs) have been highlighted as a key resource to help inform clinicians and health organizations about current evidence and improve the quality of care [4–6].

The existence of CPGs, however, does not ensure evidence-based care is routinely delivered. Clinicians may be unfamiliar with current CPGs, and some recommendations may conflict with clinical training or experience [7]. Clinicians may lack trust in CPGs due to insufficient transparency in reporting methods and concerns about quality [8–10]. Health organization characteristics and processes can also positively or negatively influence the extent to which CPGs are followed [11, 12].

CPGs are informed by subject matter experts and careful review of current scientific evidence, typically resulting in formal recommendations for clinical decision-making and care delivery [13]. Current CPG recommendations differ from traditional management approaches for LBP. For example, The American College of Physicians (ACP) recommends non-pharmacological care as the first option for people with LBP [14]. Several non-pharmacological therapies, such as exercise and various manual therapies, demonstrate similar benefits to pharmacological therapies with comparatively less risk [15–23] and lower healthcare costs [4–6].

Unfortunately, there is little evidence that CPG-recommended care for LBP is prevalent across health systems [24, 25]. Some evidence suggests treatments of unknown value and other high-cost, low-value care for LBP are increasing [26, 27]. Health organizations and providers play a vital role in guideline-concordant care. Therefore, providers caring for patients with LBP are essential stakeholders who offer key insight into applying clinical guidelines to clinical decision-making, diagnosis, treatment decisions, and referral pathways. Academic health centers, consistent with other health systems [28, 29], are determined to facilitate guideline-concordant care by creating care pathways aligned with guideline recommendations for LBP. This study aimed to develop an evidence-based care pathway for LBP for use in an academic health center and validated by a multidisciplinary panel of provider stakeholders.

## Methods

We used a three-step process to accomplish study objectives. First, we worked with a librarian to conduct bibliographic searches in MEDLINE, EMBASE, CINAHL. The search strategy focused on LBP CPGs, systematic reviews focused on evidence-based care for LBP, and clinical trials published after the most recent systematic reviews. Authors (GB, DPD, and CG) independently conducted title/abstract and full text screening to identify relevant studies. The authors independently abstracted study characteristics and key findings from systematic reviews and clinical trials or recommendations for CPGs. We used these data, with an emphasis on CPG recommendations, to develop the seed statements. Second, we developed seed statements as individual components of a care pathway designed to support and facilitate the clinical application of evidence-based care for LBP. Seed statements were designed to address consistency in care delivery regardless of health profession and whether the patient presented with acute or chronic low back pain. Third, we recruited a multidisciplinary panel of Duke University Hospital System providers who care for patients with LBP (e.g., primary care, physical therapy, physiatrists, orthopedic surgery, chiropractic) to review and validate seed statements using modified Delphi methodology. This study was reviewed and found exempt by the Duke University Health System Institutional Review Board (IRB protocol number: Pro00109618).

## Source document and seed statements

One investigator (RV), an experienced clinician, educator, and clinical researcher, initially developed seed statements. Other investigators (GB, DPD, CG, HG, JM) refined statements through an iterative process and facilitated statement formatting for Delphi panel review (GB, DPD). Topic areas included LBP assessment, referral pathways, and general clinical management. The investigative team then further reviewed, refined, and organized draft statements into thematic headings. Twenty-one statements were distributed to Delphi panelists for the initial review round.

## Modified Delphi consensus process

Consensus was conducted using a modification of the RAND Corporation/University of California, Los Angeles methodology on appropriateness ratings [30]. Data were collected electronically using Qualtrics (v. 2020; Qualtrics, Provo, UT). Panelists were invited if they were providers with the Duke University Health System who commonly treat LBP and in the following disciplines: orthopedic and neurosurgery, primary care, physiatry, osteopathic medicine, physical therapy, nurse practitioner, physician assistant, and chiropractic. Panelists from these disciplines represented the majority of provider types who cared for people with LBP within the Duke University Health System and for whom the clinical care pathway for LBP was most applicable. A Qualtrics link containing an overview of the project, the purpose of recommendations included in seed statements, instructions on participating in the Delphi panel, participants’ expectations, and approximate time required to participate were sent to 44 potential participants via E-mail. Interested participants signed an electronic consent form before initiating the survey.

Demographic characteristics collected from panelists included profession, age, employment duration at Duke, race, and ethnicity. Panelists individually rated the content of each seed statement using the following ordinal scale: 1–3 “highly inappropriate,” 4–6 “undecided,” or 7–9 “highly appropriate.” Each seed statement contained text fields to provide an opportunity to comment on any statement rated below 7. Each statement also included embedded references supporting the statements, with electronic links to PubMed abstracts.

After the panelist review, two investigators (GB and DPD) entered the de-identified numerical ratings and panelist comments into Microsoft Excel. Consensus was defined as a minimum of 80% of participants rating a single seed statement as “highly appropriate” (a rating of 7–9). Statements not reaching consensus were revised and reassessed by panelists until consensus was obtained or the statement was removed. The consensus process took place between March and October 2022. The first round of seed statement review began in March 2022, lasting four weeks. The second round started in October 2022, lasting two weeks and consisting only of revised statements that did not reach consensus during the first review round.

## Result

Thirty-five panelists rated seed statements. Demographic characteristics are reported in Table 1. Participants were predominantly male (*n* = 24, 68.6%) with a mean (SD) age of 44.8 (14.5). Members of several disciplines participated in the Delphi panel. The most commonly reported degrees were medicine (MD) or Osteopathic medicine (DO) (*n* = 22, 62.9%). The majority of participants identified as physicians/clinicians (*n* = 31, 73.8%) working in several different areas such as neurosurgery (*n* = 13, 36.1%), orthopedics (*n* = 9, 25.7%), emergency medicine (*n* = 5, 14.3%) and physical therapy (*n* = 4, 11.4%). The largest single group of participants were employed at Duke for 4 to 10 years (*n* = 16, 48.5%) and in their professions for over ten years (*n* = 15, 44.1%). After the first review round, 20 of 21 statements met the a-priori definition of consensus. The statement failing to reach a consensus was then revised into two separate statements based on panelist feedback. Eleven participants responded to the follow-up survey (31.4% response rate).

**Table 1:** Demographic characteristics of Delphi panelists (*n *= 35)

Characteristics	*n* (%)
Age—mean (SD)	44.8 (14.5)
*Gender*	
Male	24 (68.6)
Female	11 (31.4)
Clinical degree	
Medical Doctor/Doctor of Osteopathic *Medicine*	22 (62.9)
Doctor of Physical Therapy	8 (22.9)
Physician Assistant	4 (11.4)
Doctor of Chiropractic	1 (2.9)
*Clinical Department*	
Neurosurgery	13 (37.1)
Orthopedic	9 (25.7)
Emergency Medicine	5 (14.3)
Physical Medicine & Rehabilitation	4 (11.4)
Physical Therapy	4 (11.4)
Primary Care (Family or Internal Medicine)	1 (2.9)
*Occupation Level*	
Clinician/Physician	31 (73.8)
Professor/Educator	7 (16.8)
Researcher	3 (7.1)
Other	1 (2.3)
*Length of employment at Duke*^*a*^	
0 to 3 years	9 (27.3)
4 to 10 years	16 (48.5)
More than 10 years	8 (24.2)
*Years in profession*^*a*^	
0 to 3 years	6 (17.7)
4 to 10 years	13 (38.2)
More than 10 years	15 (44.1)

^a^ *n* = 33

Twenty-one seed statements comprised topics relevant to evidence-based clinical management of LBP, including processes associated with diagnosis, diagnostic imaging, treatment approaches for initial and specific presentations, and referral pathways. Seed statements and panelist ratings are reported in Table 2. The single statement that did not reach consensus during the initial review was: “Secondary pharmacological management should include Tramadol or Duloxetine.” Panelist comments included an unwillingness to prescribe Tramadol, preferring non-opiate muscle relaxants, and a lack of conclusive evidence supporting duloxetine as a safe and effective therapy with a low risk for dependence.

**Table 2:** Results of initial seed statement review

Seed statement	Agreement (Rated as 7–9, or “highly appropriate”)
*Patients seeking initial care from Duke Health for LBP may be evaluated by different provider disciplines and points of entry such as the emergency room, primary care, orthopedics, physiatry, chiropractic, physical therapy, and neurology. Regardless of provider discipline or point of entry, clinical evaluation of patients initiating care with any of these first access clinicians should include: (The pretext header was attached to the following two seed statements)*	
A detailed clinical history Including prior treatments and response, prior related symptoms or concerns, general health history, and review of systems	30/30 (100%)
Assessment/screening for serious or systemic disease, such as progressive neurological deficit, infection, abdominal aortic aneurysm, renal disease, primary tumors or metastatic disease	29/30 (96.7%)
For patients seeking initial care from Duke Health, imaging studies should be performed only when clinical history or symptoms suggest possible serious disease wherein imaging offers key information to rule in / out clinical suspicions or inform urgent/emergent care or other specialty management	28/30 (93.3%)
After screening for serious or systemic disease, the first access clinician should make a clinical decision based on the patient’s presenting condition regarding whether the patient should be 1) treated by the first access clinician, 2) referred to specialty care for further evaluation, surgical consult or pain management with a physiatrist, or 3) referred to a primary spine practitioner (chiropractic or physical therapy) for conservative non-pharmacological treatment	28/30 (93.3%)
*Regardless of provider discipline, clinicians treating patients with LBP should incorporate: (The pretext header was attached to the following three seed statements)*	
Psychological, social, assessment/screening for factors that may influence prognosis and/or contribute to the problem is also appropriate	28/30 (93.3%)
Screening for relevant environmental factors is appropriate	27/20 (90.0%)
Education to inform patients about their condition with the purpose of fostering health literacy, providing reassurance, helping patients make more informed decisions about healthcare, and encouraging self-monitoring and self-management capacity	30/30 (100.0%)
Evidence of mild or moderate radiculopathy is not a stand-alone indication for advanced imaging	24/30 (80.0%)
Evidence of severe radiculopathy is an indication for advanced imaging and consultation with a spine surgeon	27/30 (90.0%)
Psychological factors can increase risk for developing chronic LBP or reduce a prognosis for recovery	29/29 (100.0%)
When psychological factors are present, providers caring for patients with LBP should employ education and consider co-management with others skilled in therapies designed to directly address them, such as cognitive behavioral therapy, and acceptance and commitment therapy	28/29 (96.6%)
The American College of Physicians recommend nonpharmacological treatment for the initial course of care for patients with acute LBP that is not associated with pathology. Evidence-based, initial nonpharmacological care for acute LBP may include: • Superficial heat • Manual therapies such as therapeutic massage • Acupuncture • Spinal manipulation (thrust and non-thrust passive joint mobilization procedures) • Exercise (e.g., tai chi and yoga)	30/32 (93.8%)
Patients with LBP that does not respond favorably to nonpharmacological therapies should initially be referred for evaluation and management for guideline-concordant conservative pharmacotherapy by a primary care provider	26/29 (89.7%)
Initial pharmacological management should consist of nonsteroidal anti-inflammatory drugs, or tramadol or duloxetine as second-line therapy	27/29 (93.1%)
Secondary pharmacological management should include tramadol or duloxetine	20/29 (69.0%)*
Patients with LBP that does not respond favorably to conservative pharmacotherapies should be referred for evaluation by an interventional spine specialist or pain management specialist	26/29 (89.7%)
Patients with LBP that does not respond favorably to interventional spine or pain management approaches should be referred for evaluation or an e-consult by a spine surgeon to determine if evidence-based criteria for surgical intervention are met	26/29 (89.7%)
There may be circumstances when specific factors warrant a deviation from the general referral pathway model, such as when progressive neurological deficit is present, indicating urgent spine surgical consult rather than first referring for a trial of care in pain management	29/29 (100.0%)
Step 1: Conduct a screening evaluation for serious or systemic disease. If serious or systemic disease is identified, refer for appropriate specialty care. If not, proceed to step 2	29/29 (100.0%)
Step 2: For patients without serious of systemic disease, treat with guideline recommended nonpharmacological therapies incorporating biopsychosocial components or refer to a provider who offers this approach and comanage with other providers as clinically indicated	27/29 (93.1%)
Step 3: For patients unresponsive to treatment, consider whether additional co-management or an alternate treatment is viable. If an additional trial of care is appropriate, initiate additional trial. If not, refer to a provider with the next most conservative approach unless otherwise indicated	28/29 (96.6%)

*Indicates consensus was not reached

Panelist comments suggested low ratings related to a single statement addressing two medications with different classifications, Tramadol and Duloxetine. Therefore, the statement was revised into two statements for a second review. In the second round, consensus was reached on one revised statement and failed to reach consensus on one revised statement (Table 3). The statement failing to reach consensus was “Secondary pharmacological management may consist of Duloxetine, when tolerated and in the absence of contraindication(s).” Although the statement was consistent with ACP guidelines, panelists did not reach a consensus on appropriateness due to remaining questions about Duloxetine’s efficacy (e.g., whether it functions primarily as a pain or antidepressant agent, concerns for dependency, and lack of experience with prescribing). Therefore, the statement was removed from the final care pathway.

**Table 3:** Results of secondary seed statement review

Revised seed statement	Agreement (Rated as 7–9, or “highly appropriate”)
Secondary pharmacological management may consist of tramadol, when tolerated and in the absence of contraindication(s)	9/11 (82.0%)
Secondary pharmacological management may consist of duloxetine, when tolerated and in the absence of contraindication(s)	7/11 (64.0%)*

*Indicates consensus was not reached

**Fig. 1:**
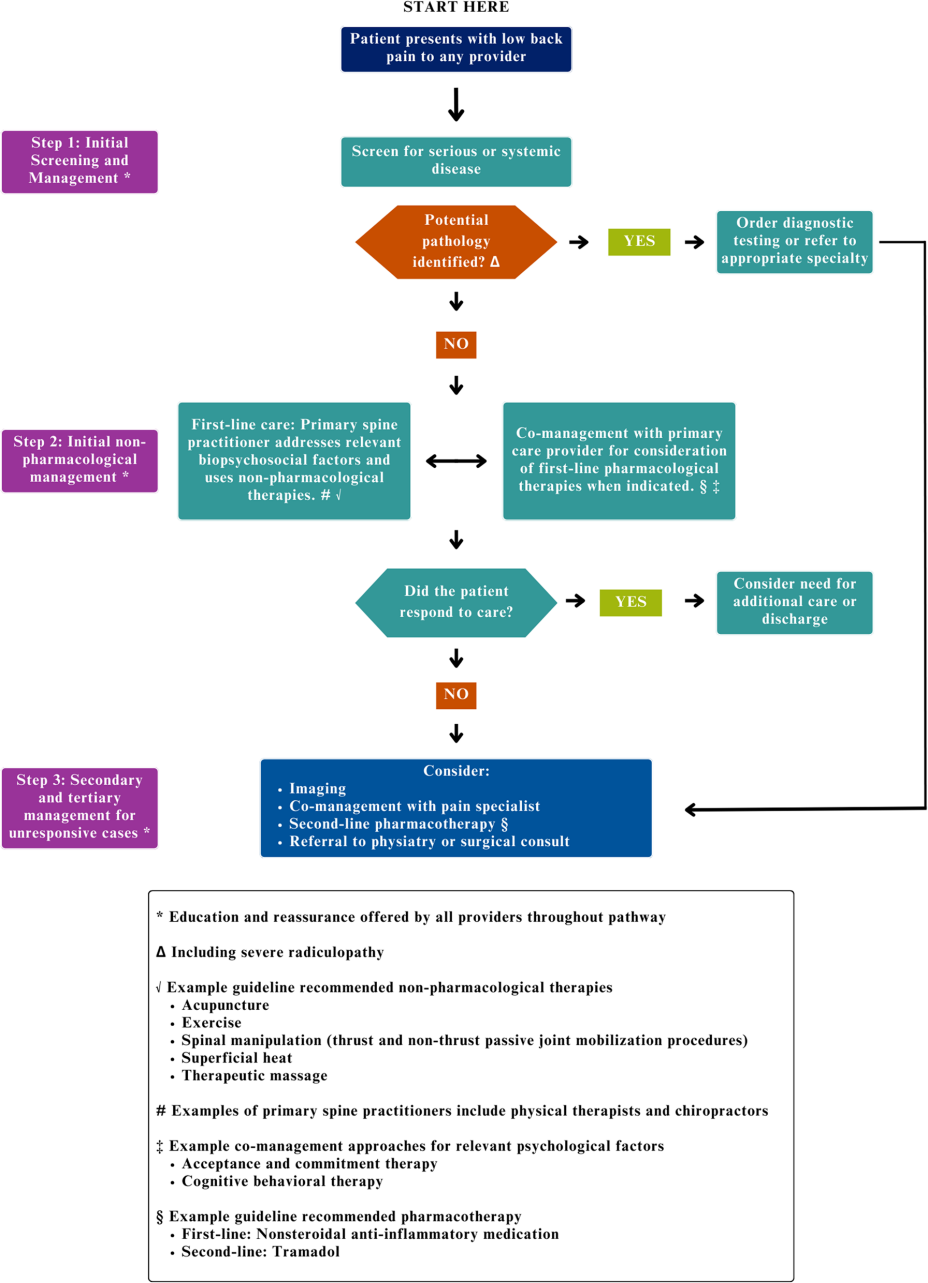
Final care pathway for LBP

Seed statements reaching consensus were used to develop the final care pathway (Fig. 1). The pathway describes general management processes beginning with a patient presenting with LBP. The first significant clinical management process indicated is an evaluation for red flags that suggest urgent referral to the emergency department or a surgeon for appropriate imaging and treatment. If patients do not present with red flags, they should be referred to a primary spine practitioner (PT or DC) for a clinical examination consisting of patient history, physical, and psychosocial examination. After clinical examination, treatment should consist of non-pharmacological care unless there is evidence of serious pathology, or severe radiculopathy (e.g., identified by reduced/absent nerve root signaling) is present. When a trial of non-pharmacological care is ineffective, referral for pharmacological treatment should be considered, followed by referral for evaluation by pain management specialist when there is a limited/no response. Finally, referral and consult with a spine surgeon is appropriate to determine if evidence-based criteria for surgical intervention are met in cases when there is a limited/no response to care.

## Discussion

While prior LBP care pathways have been developed within academic health centers in Europe, to our knowledge, this is the first Delphi consensus process conducted to develop a LBP care management pathway using a multidisciplinary panel of providers with experience caring for patients within a US academic health system [31, 32]. Our findings are consistent with existing CPGs, [14, 33, 34] suggesting that the core components of the care pathway developed in this study may have broader applicability for other healthcare settings.

Participants arrived at consensus for 21 seed statements across two rounds, while one statement regarding the use of Tramadol and Duloxetine required three rounds to achieve consensus. Consistent with our findings that clinicians were less likely to agree with CPG recommendations regarding use of these two medications, there is considerable uncertainty regarding optimal pharmacological therapy for LBP [35]. Such uncertainty highlights the need for additional high-quality randomized controlled trials of pharmacological therapies for LBP to better inform CPGs and their resultant care pathways.

Core to the care pathway developed under this initiative is an acknowledgment that members of different health professions can appropriately conduct diagnostic evaluations for LBP. In summary, regardless of discipline, clinical evaluation should include a detailed history, red flag screening, and psychosocial screening. Consistent with CPGs from the American College of Radiology, initial evaluation of LBP typically should not include imaging unless red flags suggest serious underlying pathology [36]. Following diagnostic evaluation, first-line care is initiated by primary spine practitioners offering nonpharmacological care, with psychological co-management when indicated [37, 38]. When patients do not respond favorably, pharmacological therapy is considered, followed by interventional pain management, and finally, surgical evaluation if appropriate criteria are met.

Though evidence-based care pathways may be applicable in a given setting, improved outcomes do not necessarily follow. Three recent clinical trials compared risk-stratified care using the STarT Back tool with usual care for participants with LBP [39–41]. Each trial reported similar outcomes for both groups, suggesting referral pathways informed by risk stratification added no clinical benefit. However, two studies showed limited fidelity in implementing risk-stratified care. We attempted to address issues of implementation by working with an interdisciplinary team to conceptualize this project and using the Delphi process to incorporate input from clinicians across the broad range of disciplines for which the care pathway was intended. These methods leveraged the practical knowledge of providers, theoretically facilitating acceptance and laying a foundation for successful implementation.

The next logical step in this line of research is to assess the feasibility of implementation and, subsequently, the clinical effectiveness of pathway-driven care for low back pain. Consistent with this line of research, the academic health systems of Duke University, Dartmouth College, and the University of Iowa have recently begun recruiting participants for an NIH-funded pragmatic clinical trial designed to evaluate outcomes associated with asking patients to consider seeing a physical therapist or doctor of chiropractic before seeking care from a primary care physician [42].

This study has several important strengths. For example, seed statements were informed by current evidence, notably CPGs relevant to management for LBP. Further, we recruited experienced providers across disciplines (e.g., Medicine, Osteopathic Medicine, Physician Assistant, Physical Therapy, and Chiropractic) and care settings (e.g., emergency, orthopedics, neurosurgery, physical medicine and rehabilitation, and primary care), allowing for a broad perspective on best practices for clinical care. We also acknowledge several important limitations. We did not include professionals from every conceivable clinical discipline that may engage patients with LBP (e.g., pain psychologists). In addition, panelists in our study were all employed at one academic medical center, which could limit the extrapolation of findings to other settings. Finally, studies of this type are always limited by those who did and did not participate.

## Conclusion

Healthcare providers across various disciplines within a large academic health center supported statements interpreting current clinical practice guidelines for low back pain care. This study represents a step toward supporting and coordinating guideline-concordant care among multiple health disciplines. Additional research is needed to assess how the care pathway developed in this study influences clinical management and outcomes. Fortunately, such investigation is already being initiated through the NIH Collaboratory.

## Data Availability

The datasets used and analyzed during the current study are available from the corresponding author upon reasonable request.

## Abbreviations

ACP: American College of Physicians
CPGs: Clinical practice guidelines
LBP: Low back pain

## Declarations

### Data statement

The authors will provide the data for this study under reasonable request.
